# Thyroid Functions and Cognitive Decline in the Elderly

**DOI:** 10.7759/cureus.74484

**Published:** 2024-11-26

**Authors:** Ayaz Muhammad, Ijaz Ul Haq, Mehr Ali Khan, Hafiz Syed Ahmad Hassan, Bilal Aman, Muhammad Muneeb Arshad

**Affiliations:** 1 Internal Medicine, Mardan Medical Complex, Medical Teaching Institution, Mardan, PAK; 2 Internal Medicine, Nenagh General Hospital, Tipperary, IRL; 3 Medicine, Northwest General Hospital and Research Center, Peshawar, PAK; 4 Pulmonology, Ayub Teaching Hospital, Abbottabad, PAK; 5 Medicine, Services Hospital Lahore, Lahore, PAK; 6 Medicine, Waterford Regional Hospital, Waterford, IRL; 7 Medicine, Mardan Medical Complex/Bacha Khan Medical College, Mardan, PAK; 8 Internal Medicine and Cardiology, University Hospital Birmingham NHS foundation Trust, Birmingham, GBR

**Keywords:** cognitive decline, elderly, free t4, mmse, moca, thyroid function, tsh

## Abstract

Background

The maintenance of cognitive health depends on thyroid hormones, and it is becoming more widely acknowledged that thyroid hormone issues may be a factor in cognitive decline in the aged.

Objective

This study aimed to investigate the association between thyroid hormone levels and cognitive decline among elderly individuals, considering the influence of age-related factors and comorbidities.

Methodology

Over the course of two years, 218 adults 60 years of age and older with clinically diagnosed hypothyroidism or subclinical thyroid disease were included in a prospective observational research. Serum levels of thyroid-stimulating hormone (TSH), free thyroxine (T4), and free triiodothyronine (T3) were measured at baseline and at 6, 12, 18, and 24-month intervals to evaluate thyroid function. The Mini-Mental State Examination (MMSE) and the Montreal Cognitive Assessment (MoCA) were used to assess cognitive function at the same intervals. Factors linked to cognitive deterioration were identified using multivariate regression analysis.

Results

The study found that TSH levels decreased from 3.20 ± 1.82 µIU/mL at baseline to 2.80 ± 1.51 by 24 months (p < 0.001), while free T4 levels increased from 13.50 ± 2.53 pmol/L to 14.20 ± 2.52 pmol/L (p = 0.020). Cognitive scores declined significantly, with MMSE scores dropping from 23.27 ± 4.64 to 21.80 ± 4.89 (p = 0.005) and MoCA scores from 21.20 ± 5.11 to 20.03 ± 5.51 (p = 0.012).

Conclusion

The results show a strong correlation between thyroid malfunction and cognitive loss in the elderly, emphasizing the need to closely monitor thyroid function to maintain cognitive function in this population.

## Introduction

Age, genetics, chronic illnesses, lifestyle, mental health, and sleep quality are some of the factors that contribute to cognitive decline, characterized by progressive declines in memory, attention, and problem-solving abilities [1]. Because they promote neuron health and neuroprotection, thyroid hormones, which are essential for metabolism and brain health, have a big impact on cognitive performance. Cognitive decline in older persons can be made worse by thyroid malfunction, especially hypothyroidism, which has been linked to symptoms including mood swings, exhaustion, and memory loss [2]. Thyroid function monitoring may assist address a significant component associated with cognitive decline and perhaps promote older adults' cognitive health, particularly in ageing populations [3].

Thyroid hormones are essential for metabolism, growth, and development, among other physiological functions [4]. The relationship between thyroid function and cognitive functioning in older adults has drawn more attention [5]. Cognitive abilities including memory, attention, and executive function may be impacted by changes in thyroid hormone levels as people age; these changes are often characterized by hypothyroidism or subclinical thyroid dysfunction. A higher risk of dementia and other neurodegenerative diseases may result from these changes, which can cause significant reductions in cognitive function [6].

According to recent research, there may be a connection between thyroid hormone levels and cognitive decline, and even minor thyroid malfunction may have a negative impact on cognitive performance [7]. The thyroid gland's hormones are essential for neurodevelopment and neuroprotection, and it controls the metabolism of almost all bodily cells [8]. Age-related cognitive loss may be made worse by disturbances in this delicate equilibrium, perhaps as a result of factors such as decreased neurogenesis, changed neurotransmitter levels, and elevated oxidative stress [9].

Additionally, additional variables that are common in older persons, such as comorbidities, medication usage, and lifestyle factors, may confuse the link between thyroid function and cognitive impairment [10]. In order to better understand the underlying processes, it is crucial to detangle these interactions since these factors have the potential to either disguise or intensify the impact of thyroid disease on cognitive health [11].

Investigating the complex link between thyroid function and cognitive health is essential, especially in light of the growing number of older people worldwide and the increasing incidence of cognitive impairment. In addition to determining the degree to which thyroid disease affects cognition, this study intends to guide future screening and treatment strategies for optimizing cognitive function in older adults.

Research objective

To examine the association between thyroid hormone levels and cognitive decline in the elderly, considering the influence of age-related factors and comorbidities.

## Materials and methods

Study design and setting

This study utilized a prospective observational design conducted at Ayub Teaching Hospital Complex, Abbottabad, over a two-year period from January 2019 to December 2020.

Inclusion and exclusion criteria

Participants in the research were to be at least 60 years old and have either subclinical thyroid impairment, as shown by blood thyroid hormone levels, or clinically diagnosed hypothyroidism. In order to participate in the research, participants had to be willing and able to provide informed consent. People with a history of major neurological conditions (like stroke, traumatic brain injury, or severe dementia) that could affect cognitive function on their own, individuals receiving treatment for thyroid dysfunction (such as hormone replacement therapy or antithyroid drugs), people with serious mental illnesses, and people with recent acute illnesses or infections that could confuse cognitive evaluation were all excluded.

Sample size

The World Health Organization (WHO) formula for estimating proportions was used to determine the necessary sample size for this study. Based on a 15% expected prevalence of thyroid dysfunction in the elderly population and a 5% margin of error, the initial estimate of 198 participants was obtained. The final corrected sample size, which took into consideration a 10% dropout rate, was found to be 218 individuals. Despite potential attrition, this modification made sure the research had sufficient statistical power.

Data collection

Standardized questionnaires and clinical exams were used in a thorough approach to data collection. Serum levels of thyroid-stimulating hormone (TSH), free thyroxine (T4), and free triiodothyronine (T3) were measured at baseline and at follow-ups at 6, 12, 18, and 24 months to assess thyroid function. The Mini-Mental State Examination (MMSE) and the Montreal Cognitive Assessment (MoCA) were used to measure cognitive performance at the same time intervals during follow-up.

A standardized questionnaire was used to collect comprehensive information on participants' medical history, comorbidities, prescription usage, demographic details, and lifestyle factors such as physical activity and dietary preferences (see Appendices). Dietary habits were specifically assessed to determine adherence to a balanced diet, which was defined as one that includes appropriate proportions of macronutrients (carbohydrates, proteins, and fats) and essential micronutrients (vitamins and minerals). The balanced diet emphasized nutrient-dense foods like fruits, vegetables, whole grains, lean proteins, and healthy fats, with adequate hydration defined as a daily water intake of 1.5-2 liters, adjusted for medical conditions such as heart or kidney disease. The questionnaire also recorded specific dietary considerations, such as low-sodium diets for hypertension and low-glycemic foods for diabetes, and restrictions on processed foods, added sugars, saturated fats, excessive caffeine, and alcohol. Participants were asked to report their dietary habits through this standardized questionnaire, and based on their responses, they were categorized as following either a balanced or unbalanced diet. The majority of participants in our study followed a mixed diet, incorporating both plant-based and animal-based foods.

Follow-up was conducted exclusively through face-to-face visits, where participants were invited to return to the study site for assessment at the designated follow-up intervals. Participants were reminded of their upcoming visits through telephone calls, and those who missed a scheduled visit were contacted and rescheduled. To ensure accurate follow-up, participants were required to provide contact information (such as a mobile number) at the time of enrollment to facilitate appointment reminders and rescheduling. The standardized data collection form was updated to include details on follow-up visits, documenting the date, attendance, and any issues related to participant availability. This method ensured that data collection remained consistent and minimized attrition.

Statistical analysis

Statistical Package for Social Sciences (SPSS) 26 (IBM Corp., Armonk, USA) was used to examine the data. While inferential statistics evaluated the relationship between thyroid hormone levels and cognitive deterioration, descriptive statistics provided an overview of baseline characteristics. Confounding factors including age, sex, comorbidities, and medication usage were taken into account via multivariate regression analysis. Using repeated measures of ANOVA, changes in thyroid function and cognitive scores across the follow-up periods were examined. Statistical significance was defined as a p-value of less than 0.05.

Ethical statement

The study adhered to the principles of the Declaration of Helsinki and received ethical approval from the Institutional Review Board of Ayub Teaching Hospital Complex, Abbottabad (Approval No. ATH/EC-1/19) on January 13, 2019. Informed consent was obtained from all participants prior to enrollment, ensuring their right to withdraw from the study at any time without any impact on their medical care.

## Results

The demographic and baseline characteristics of the 218 research participants, whose average age was 75.20 ± 7.50 years, are summarized in Table 1. There were 118 females (54.13%) and 100 men (45.87%), making the gender distribution about equal. There was a wide range of educational attainment: 18.35% had no formal education, 32.11% had gone to school, 27.52% had some college, and 22.02% had graduated from college. In terms of diet, 98 individuals (44.95%) did not follow a healthy diet, while 120 people (55.05%) did. Of the individuals, 90 (41.28%) were physically active on a regular basis, while 128 (58.72%) mostly followed a sedentary lifestyle. None of the participants were receiving thyroid-specific therapy, although 105 individuals (48.17%) were using non-thyroid drugs. Of the individuals, 80 had hypertension (36.70%), 45 had diabetes (20.64%), and 30 had cardiovascular disease (13.76%). In addition, the participants had the following other medical conditions: 26 (11.92%) had chronic respiratory disorders, 14 (6.42%) had a history of previous surgeries, 11 (5.04%) had minor neurological issues, and 22 (10.09%) had gastrointestinal illnesses.

**Table 1:** Demographic and Baseline Characteristics of Participants

Variable		Number of Patients (n;%)
Age (years)	Mean ± SD	75.20 ± 7.50
Gender	Male	100 (45.87)
	Female	118 (54.13)
Education Level	No formal education	40 (18.35)
			School	70 (32.11)
	College	60 (27.52)
	University	48 (22.02)
	Diet	Balanced diet adherence	120 (55.05)
Unbalanced diet		98 (44.95)
Physical Activity	Regular physical activity	90 (41.28)
	Sedentary lifestyle	128 (58.72)
Medication Use	Thyroid medication	0 (0.00)
	Other medications	105 (48.17)
Comorbidities	Hypertension	80 (36.70)
	Diabetes	45 (20.64)
	Cardiovascular disease	30 (13.76)
Other Medical Conditions	Chronic respiratory disorders	26 (11.92%)
	Previous surgeries	14 (6.42%)
	Minor neurological issues	11 (5.04%)
	Gastrointestinal illnesses	22 (10.09%)

**Figure 1:**
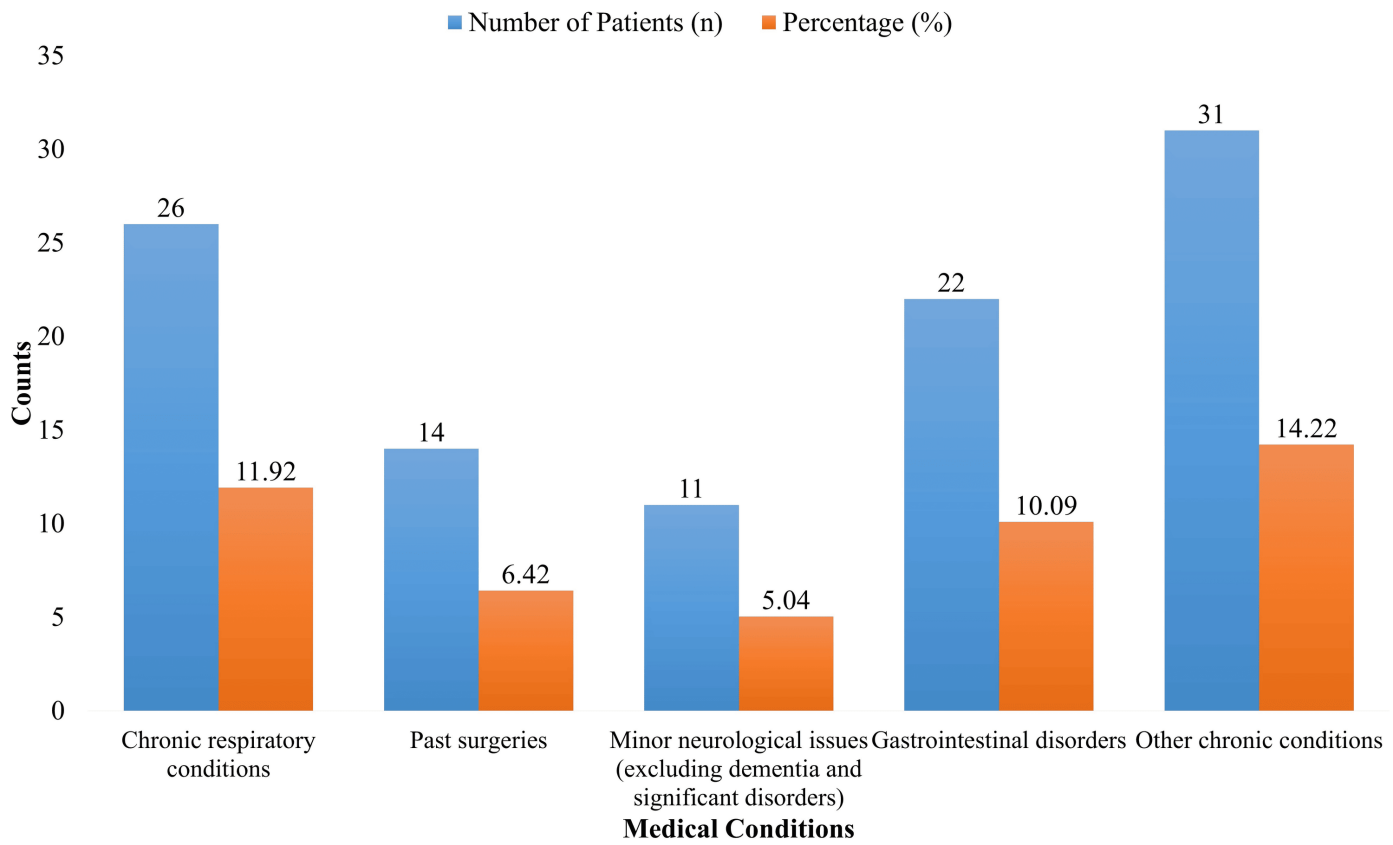
Medical Conditions of Participants

The distribution of different medical problems in the research group is shown in Figure 1. Of the 218 patients, 14 (6.42%) had a history of surgery, and 26 (11.92%) had chronic respiratory disorders. Eleven subjects (5.04%) had minor neurological problems, excluding dementia and severe neurological illnesses. Of the participants, 31 (14.22%) had various chronic health issues, and 22 (10.09%) had gastrointestinal illnesses.

The individuals' mean thyroid function and cognitive test results during a two-year period are shown in Table 2. The average TSH level was 3.20 ± 1.82 µIU/mL at baseline, and at the 24-month point, it had dropped to 2.80 ± 1.51 (p < 0.001). Over the same time period, free T4 levels rose marginally from 13.50 ± 2.53 pmol/L to 14.20 ± 2.52 pmol/L (p = 0.020). Beginning at 3.10 ± 0.83 pmol/L and increasing to 3.30 ± 0.84 pmol/L (p = 0.150), free T3 levels showed no change. Cognitive performance, as measured by the MMSE and MoCA, declined significantly; the MMSE score decreased from 23.27 ± 4.64 to 21.80 ± 4.89 (p = 0.005), while the MoCA score dropped from 21.20 ± 5.11 to 20.03 ± 5.51 (p = 0.012). The MMSE and MoCA assess cognitive function, with the MMSE focusing on memory, attention, and orientation (a score below 24 suggests decline) and the MoCA is a more sensitive test for detecting mild cognitive impairment, covering additional cognitive domains such as executive function and visuospatial skills, with a score below 26 suggesting possible impairment. Both scores decreased over 24 months, indicating cognitive decline during the study period (Table 2).

**Table 2:** Cognitive Scores and Thyroid Function Across Follow-Up Periods Measurements were taken as follows: Thyroid-Stimulating Hormone (TSH, µIU/mL), Free Thyroxine (Free T4, pmol/L), Free Triiodothyronine (Free T3, pmol/L), and cognitive function scores using the Mini-Mental State Examination (MMSE) and the Montreal Cognitive Assessment (MoCA).

Variable	Baseline (Mean ± SD)	6 Months (Mean ± SD)	12 Months (Mean ± SD)	18 Months (Mean ± SD)	24 Months (Mean ± SD)	p-Value (24 Months)
Thyroid-Stimulating Hormone (TSH, µIU/mL)	3.20 ± 1.82	3.15 ± 1.72	3.10 ± 1.63	2.95 ± 1.57	2.80 ± 1.51	< 0.001
Free T4 (pmol/L)	13.50 ± 2.53	13.70 ± 2.64	13.90 ± 2.51	14.10 ± 2.63	14.20 ± 2.52	0.020
Free T3 (pmol/L)	3.10 ± 0.83	3.15 ± 0.75	3.20 ± 0.81	3.25 ± 0.79	3.30 ± 0.84	0.150
Mini-Mental State Exam (MMSE) Score	23.27 ± 4.64	23.03 ± 4.51	22.80 ± 4.72	22.50 ± 4.85	21.80 ± 4.89	0.005
Montreal Cognitive Assessment (MoCA) Score	21.20 ± 5.11	21.00 ± 5.06	20.80 ± 5.23	20.50 ± 5.32	20.03 ± 5.51	0.012

**Figure 2:**
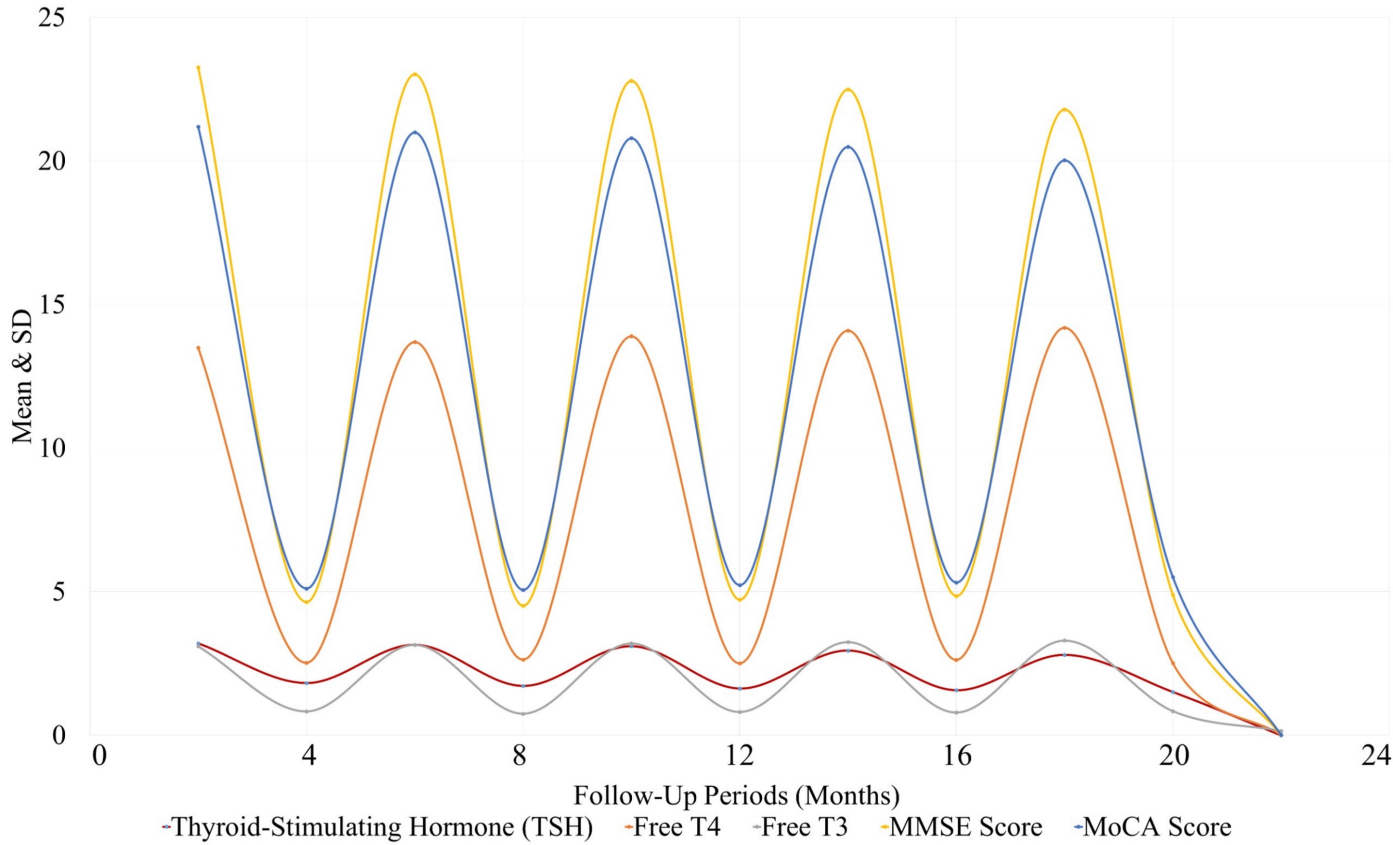
Mean Comparison of Variables Across Follow-Up Periods T3: triiodothyronine; T4: thyroxine; MMSE: Mini-Mental State Examination; MoCA: Montreal Cognitive Assessment

Figure 2 illustrates the changes in thyroid function and cognitive performance over a 24-month period in the study participants. The graph highlights the decline in TSH levels (p < 0.001), while free T4 levels show a modest increase (p = 0.020). However, free T3 levels remained relatively stable (p = 0.150). Cognitive performance, as measured by the MMSE and MoCA, showed a significant decline over time, with both tests indicating cognitive deterioration (MMSE: p = 0.005, MoCA: p = 0.012). These results suggest that, while thyroid function slightly improved in some aspects, cognitive decline was observed throughout the follow-up period.

Table 3 displays the β coefficients, p-values, and confidence intervals (CI) for a number of variables that may impact participants' cognitive deterioration. According to the research, cognitive decline is substantially correlated with greater levels of Free T4 (β = 0.33, 95% CI: 0.10 to 0.56, p = 0.003) and lower levels of TSH (β = -0.45, 95% CI: -0.68 to -0.22, p < 0.001). With a negative correlation (β = -0.50, 95% CI: -0.72 to -0.28, p < 0.001), age also became a significant factor. Cognitive health was favorably connected with both regular physical activity (β = 0.25, 95% CI: 0.02 to 0.48, p = 0.03) and education level (β = 0.40, 95% CI: 0.25 to 0.55, p < 0.001). Cognitive performance was likewise linked to balanced diet adherence (β = 0.20, 95% CI: 0.05 to 0.35, p = 0.01), although there was no significant correlation between gender and cognitive performance (β = 0.18, 95% CI: -0.05 to 0.41, p = 0.12).

**Table 3:** Multivariate Regression Analysis for Factors Associated with Cognitive Decline TSH: Thyroid-Stimulating Hormone; T3: triiodothyronine; T4: thyroxine

Variable	β Coefficient	95% CI	p-Value
TSH Level (µIU/mL)	-0.45	-0.68 to -0.22	< 0.001
Free T4 (pmol/L)	0.33	0.10 to 0.56	0.003
Free T3 (pmol/L)	0.15	-0.08 to 0.38	0.17
Age (years)	-0.5	-0.72 to -0.28	< 0.001
Gender (Female)	0.18	-0.05 to 0.41	0.12
Education Level	0.4	0.25 to 0.55	< 0.001
Regular Physical Activity	0.25	0.02 to 0.48	0.03
Balanced Diet Adherence	0.2	0.05 to 0.35	0.01
Hypertension	-0.28	-0.52 to -0.04	0.02
Diabetes	-0.35	-0.59 to -0.11	0.004
Cardiovascular Disease	-0.22	-0.45 to 0.01	0.06
Medication Use (Other Meds)	-0.18	-0.38 to 0.02	0.08
Chronic Respiratory Disorders	-0.25	-0.50 to -0.01	0.04
Previous Surgeries	-0.3	-0.55 to -0.05	0.02
Minor Neurological Issues	-0.4	-0.60 to -0.20	< 0.001

## Discussion

Research on the relationship between thyroid function and cognitive health in the elderly is crucial since cognitive impairment is becoming more common in this demographic. According to our research, thyroid function improved with time as TSH levels dropped from a baseline of 3.20 ± 1.82 µIU/mL to 2.80 ± 1.51 at 24 months (p < 0.001). This result is consistent with other research that shows that thyroid hormone levels may stabilize with proper care, perhaps preventing cognitive deterioration in the elderly [12]. Given the importance of thyroid health in maintaining cognitive function, the correlation between lower TSH levels and higher cognitive scores is strong.

The MMSE scores showed a substantial deterioration in cognitive ability, falling from 23.27 ± 4.64 at baseline to 21.80 ± 4.89 at 24 months (p = 0.005). This decrease is in keeping with other studies that found older persons with thyroid problems to have a similar pattern of cognitive decline [13,14]. Our study's MoCA results also showed a significant decline from 21.20 ± 5.11 to 20.03 ± 5.51 (p = 0.012), which lends further credence to the idea that thyroid hormone variations and cognitive health are closely related.

Furthermore, our multivariate regression analysis showed that cognitive decline was substantially correlated with lower TSH levels (β = -0.45, p < 0.001) and higher free T4 levels (β = 0.33, p = 0.003), but not with free T3 levels (β = 0.15, p = 0.17). Prior studies have shown that greater levels of T4 were linked to improved cognitive function, which supports our results [15,16]. The importance of TSH and free T4 in cognitive health is crucial because it implies that controlling thyroid hormone levels and maybe monitoring them may help manage cognitive decline.

Our findings also showed that age (β = -0.50, p < 0.001), education level (β = 0.40, p < 0.001), and physical activity (β = 0.25, p = 0.03) all had a substantial impact on cognitive outcomes, underscoring these associations. This is consistent with earlier research that suggested lifestyle choices and biological variables like thyroid hormones had an impact on cognitive resilience [17]. Therefore, including thyroid function testing in regular assessments of senior citizens' cognitive health may help lessen the impacts of cognitive decline.

Study strengths and limitations

The prospective observational design, thorough evaluation of thyroid function and cognitive functioning, and inclusion of a well-defined participant cohort aged 60 and above strengthen the reliability of this study. Standardized cognitive assessment tools, including MMSE and MoCA, further enhance the robustness of the data. To mitigate potential biases, we employed a comprehensive multivariate analysis that included not only demographic and lifestyle factors but also comorbidities such as hypertension, diabetes, and cardiovascular diseases. This allowed us to better understand the independent effects of thyroid hormone levels on cognitive decline while controlling for the influence of these conditions.

While some limitations exist, steps were taken to minimize their impact. Excluding patients with severe neurological conditions was done to ensure that thyroid dysfunction was not confounded by other significant cognitive impairments. Additionally, the study’s 24-month timeframe, while providing valuable insights into the medium-term effects of thyroid function on cognitive health, will be addressed by future studies with longer follow-up periods to capture long-term trends. Future research with larger cohorts, longer durations, and broader participant inclusion will further enhance our understanding of the complex relationship between thyroid function, comorbidities, and cognitive decline in older adults.

## Conclusions

This research underscores a strong and independent relationship between thyroid dysfunction and cognitive decline in the elderly, highlighting that lower TSH levels and higher free T4 levels are significantly associated with reduced cognitive performance. These findings emphasize the importance of monitoring thyroid function in older adults, as even subtle disruptions can negatively affect cognitive health and potentially accelerate age-related cognitive decline. Furthermore, other factors such as age, education level, physical activity, and dietary habits were shown to play critical roles, suggesting the need for a multimodal approach to cognitive preservation in this vulnerable population.

By integrating a comprehensive multivariate analysis that accounts for the influence of comorbidities such as diabetes, hypertension, and cardiovascular disease, this study strengthens its conclusion that thyroid dysfunction is a pivotal determinant of cognitive health. While the effects of comorbidities were found to be additive rather than moderating, the findings highlight the necessity of addressing both thyroid function and chronic conditions in a cohesive management strategy. These results support the development of targeted interventions to maintain optimal thyroid function and manage comorbidities as part of efforts to preserve cognitive abilities in older individuals. Future research with extended follow-up and larger cohorts is essential to confirm these insights and explore the underlying mechanisms linking thyroid health and cognitive decline.

## Appendices

**Table 4:** Questionnaire: Thyroid Functions and Cognitive Decline in The Elderly

Question	Response Option 1	Response Option 2	Response Option 3	Response Option 4	Other / Specify	Additional Comments
Demographic and Lifestyle						
1. Age	__ years	-	-	-	-	-
2. Gender	Male	Female	Other: _____	-	-	-
3. Education Level	No formal education	School	College	University	-	-
4. Diet Adherence	Balanced	Unbalanced	-	-	-	-
5. Physical Activity Level	Regular physical activity	Sedentary	-	-	-	-
6. Medical History (Conditions)	Hypertension	Diabetes	Cardiovascular Disease	-	Other: _____	-
7. Current Medication Use (Non-Thyroid)	Yes	No	-	-	-	-
Medical History						
8. Previous operations?	Yes	No	-	-	-	-
9. Chronic respiratory disorders?	Yes	No	-	-	-	-
10. Minor neurological issues? (excluding severe illnesses)	Yes	No	-	-	-	-
11. Other chronic health issues?	Yes	No	-	-	-	-
12. Gastrointestinal illnesses?	Yes	No	-	-	-	-
Thyroid Function						
13. Recent thyroid function test?	Yes	No	-	-	-	-
14. Most recent TSH level	Below 2.0 µIU/mL	2.0 – 3.0 µIU/mL	3.1 – 4.0 µIU/mL	Above 4.0 µIU/mL	-	-
15. Typical Free T4 level	Below 13.0 pmol/L	13.1 – 15.0 pmol/L	Above 15.0 pmol/L	-	-	-
16. Typical Free T3 level	Below 3.0 pmol/L	3.1 – 3.5 pmol/L	Above 3.5 pmol/L	-	-	-
Cognitive Function						
17. Frequency of memory issues	Never	Rarely	Occasionally	Frequently	-	-
18. Difficulty in cognitive areas (select all that apply)	Memory	Attention	Problem-solving	Language	Visual-spatial skills: _____	-
19. Mini-Mental State Exam (MMSE) taken?	Yes	No	-	-	-	-
20. Most recent MMSE score	Below 20	20-25	26-30	-	-	-
21. Montreal Cognitive Assessment (MoCA) taken?	Yes	No	-	-	-	-
22. Most recent MoCA score	Below 20	20-25	26-30	-	-	-
23. Cognitive change over last 12 months	Improved	Stayed the same	Declined	I’m not sure	-	-
24. Difficulty in daily tasks due to cognitive issues?	Yes, much harder	Yes, slightly harder	No change	Easier	-	-
